# Development of a novel intramuscular liposomal injection for advanced meloxicam delivery: Preparation, characterization, *in vivo* pharmacokinetics, pharmacodynamics, and pain assessment in an orthopedic pain model

**DOI:** 10.1016/j.ijpx.2024.100284

**Published:** 2024-09-14

**Authors:** Pierre A. Hanna, Hatim A. Al-Abbadi, Mohamed A. Hashem, Aziza E. Mostafa, Yasmina K. Mahmoud, Eman A. Ahmed, Ibrahim M. Hegab, Ibrahim E. Helal, Mahmoud F. Ahmed

**Affiliations:** a Department of Pharmaceutics and Industrial Pharmacy, Faculty of Pharmacy, Suez Canal University, Ismailia 41522, Egypt; b Faculty of Medicine, University Hospital, King Abdulaziz University, Jeddah 80212, Saudi Arabia; c Department of Surgery, Anesthesiology and Radiology, Faculty of Veterinary Medicine, Suez Canal University, 4.5 Ring Road, Ismailia 41522, Egypt; d Department of Pharmaceutical Analytical Chemistry, Faculty of Pharmacy, Suez Canal University, Ismailia, Egypt; e Department of Biochemistry, Faculty of Veterinary Medicine, Suez Canal University, 4.5 Ring Road, Ismailia 41522, Egypt; f Department of Pharmacology, Faculty of Veterinary Medicine, Suez Canal University, 4.5 Ring Road, Ismailia 41522, Egypt; g Department of Animal, Poultry and Fish Behavior and Management, Faculty of Veterinary Medicine, Suez Canal University, Ismailia 41522, Egypt; h Department of Agriculture, Faculty of Environmental Science, King Abdulaziz University, Jeddah 80208, Saudi Arabia

**Keywords:** Meloxicam, Liposomes, Pain management, Pharmacokinetics, Interleukin-6, CMPS-SF pain scale

## Abstract

Pain produces several physiological, and degenerative complications. This study aimed to formulate meloxicam (MLX) in liposomes to increase solubility and deliver MLX in a controlled manner to overcome its poor aqueous solubility and relatively short t_1/2_ problems. Liposomes were prepared by thin film hydration followed by ultrasonication. Tests for characterizing formulations included particle size, span, entrapment efficiency, drug loading, stability, differential scanning calorimetry (DSC), Fourier transformation infrared (FT-IR) spectroscopy, morphology, *in vitro* release, release kinetics mathematical modeling, and an *in vivo* pain model in dogs undergoing orthopedic surgeries, followed by *in vivo* pharmacokinetics, pharmacodynamics, and pain assessment studies in comparison to the reference standard, Mobitil®. Liposomal MLX had a particle size of around 100 nm, 82 % entrapment efficiency, and 4.62 % drug loading. Stability studies, DSC, and FT-IR spectroscopy indicated that liposomes were highly stable. The formulation showed an improved *in vitro* controlled release pattern and an enhanced *in vivo* pharmacokinetic behavior as manifested by higher t_1/2_ and AUC_0__–__24_ and lower Cl/F in comparison to Mobitil®. The pharmacodynamics study and pain scales demonstrated liposomal MLX managed postoperative pain better than Mobitil®. In conclusion, the incorporation of MLX in liposomes increased its solubility and stability, as well as its pain management properties.

## Graphical abstract

**Figure:**
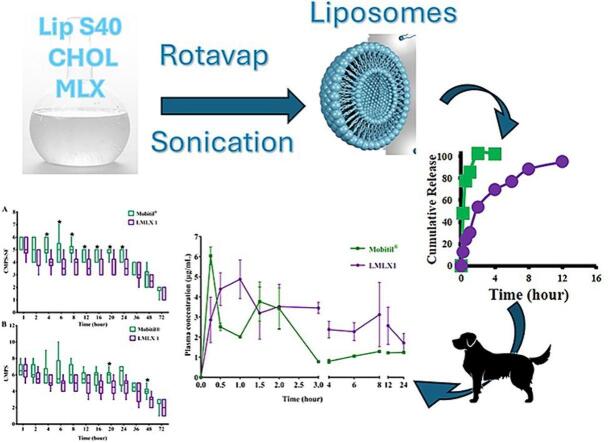

## 1 Introduction

Postoperative pain management is a crucial aspect of surgical treatment (Testa et al., 2021). Postoperative pain is the most widely experienced kind of acute pain, affecting around 80 % of surgical patients and peaking in the first few days after surgery Moraes et al., 2024). Pain may increase surgical complications, delay and hinder wound healing, contribute to persistent pain, and lengthen hospitalization (Deneuche et al., 2004). Therefore, efficient pain control contributes to a quicker recovery and restoration of normal function (Gruet et al., 2011).

Pain is mostly alleviated using non-steroidal anti-inflammatory drugs (NSAIDs) or opioids, with a preference for NSAIDs due to fewer adverse effects and restrictions compared to opioids (Paul et al., 2024; Yang et al., 2024). Meloxicam (MLX) is a member of the NSAID family with a high selectivity inhibition for cyclooxignase-2 (COX-2) that is commonly used for acute and chronic pain management (Khalil and Aldosari, 2020). MLX is highly lipophilic and belongs to Biopharmaceutics Classification System (BCS) Class II, showing a poor aqueous solubility of 7.5–15 mg/mL, making MLX solubility, absorption, and bioavailability dissolution rate-limited (Parekh et al., 2017). In light of its low aqueous solubility, it has a high t_max_, which is undesirable for treating acute pain (Yang et al., 2024). Despite its moderate biological half-life time, many patients experienced only 2–3 h of effective pain relief after post-operative MLX injection (Yang et al., 2024). This necessitates the development of a controlled release drug delivery system that releases an appreciable amount of MLX immediately followed by controlled MLX release to sustain the effect (Yang et al., 2024).

Improving the solubility of drugs with poor aqueous solubility can be accomplished through different approaches such as the co-solvent technique, solid dispersion, particle size reduction, cyclodextrin complex, pro-drug technology, co-crystallization, nanosuspension, and nanocarrier techniques (Kumari et al., 2023). Nanocarrier techniques were proven superior to conventional solubility-enhancing techniques. This is because nanotechnology has the privilege of enhancing stability, increasing shelf life, having a high carrier capacity, and controlling drug release (Kumari et al., 2023; Kwok and Chan, 2014).

Nanotechnology methods have been developed to enhance drug solubility by increasing the surface area, since dissolution is a surface phenomenon (Bhalani et al., 2022; Kwok and Chan, 2014). These nanosystems include solid lipid nanoparticles, nanoemulsions, liposomes, nanostructured lipid carriers, ethosomes, spanlastics, and niosomes (Mwema et al., 2023; Yang et al., 2024).

Liposomes are vesicles made of phospholipid bilayers that enclose an inner aqueous compartment, enabling the encapsulation of both hydrophilic and lipophilic drugs (Shah et al., 2023). Liposomes have gained recognition as an excellent drug delivery system, being used to enhance the solubility of drugs exhibiting poor aqueous solubility, regulate drug release, target drugs to specific organs or tissues, enhance drug stability, optimize drug pharmacokinetics, and increase drug bioavailability (Kumbham et al., 2023; Lee, 2020). Moreover, liposomes are well known for adopting the biphasic release required, *i.e.*, fast, immediate release followed by controlled release of the active ingredient. They were developed to enhance the antitumor activity of 5-fluorouracil (Glavas-Dodov et al., 2005) and *trans*-dehydrocrotonin (Lapenda et al., 2013) and improve the delivery of ciprofloxacin (Mehanna et al., 2009), curcumin (Fernández-Romero et al., 2018), proanthocyanidins and α-tocopherol (Zhao et al., 2024), quercetin (Jeon et al., 2015), caffeine and hydrocortisone (Wu et al., 2019), sulfanilamide (Petrović et al., 2017), letrozole, and paclitaxel (Vu et al., 2020). Regarding all these liposomal systems, the manifested release of active ingredients was characterized by fast release followed by controlled release. The release pattern and the percentage promptly released in each system differed according to the components of the liposomal nanocarrier and the rigidity of the membrane (Fernández-Romero et al., 2018; Mehanna et al., 2009; Vu et al., 2020; Wu et al., 2019).

This study aimed to formulate a liposome-loaded MLX drug delivery system for intramuscular injection with a controlled release rate of MLX to provide better drug delivery with enhanced temporal features for the management of postoperative pain accompanying orthopedic surgeries in a mongrel dog model.

## 2 Materials and methods

### 2.1 Materials

Medical Union Pharmaceutical Company (MUP, Ismailia, Egypt) kindly gifted MLX (pastel yellow solid, 99 % purity). A soy lecithin fraction with no less than 40 % phosphatidylcholine content, Lipoid S 40, was procured from Lipoid (GmbH, Germany). Cholesterol (CHOL) was obtained from Molekula Americas LLC (TX, USA). Methanol HPLC grade was procured from Fisher Scientific (NJ, USA). Chloroform was obtained from Sigma-Aldrich (Germany). Methanol was purchased from Piochem for laboratory chemicals (Giza, Egypt). Dialysis tubing cellulose membrane (molecular weight cut-off 12,000–14,000 g/mol) was procured from Sigma-Aldrich (St. Louis, USA). All other chemicals used in this study were of analytical grade.

### 2.2 Preparation of plain liposomes

Different liposomal compositions (Lipoid S40®, Lipoid S75®, and Lipoid S100®) with different concentrations of the lipid, cholesterol and drug, were tested for the preparation of liposomal carriers with best physicochemical characteristics using one factor at a time design of experiment (data not shown). Since MLX is a small molecule that is suggested to be released fast from the liposomal membrane, cholesterol was added to the formulation to increase the membrane rigidity of the liposomal nanocarriers and hence control the rate of MLX release from the nanocarrier (Haghiralsadat et al., 2018; Mehanna et al., 2009; Nie et al., 2012). Plain liposomes (PL 1 and PL 2) were prepared using the thin film hydration technique (Ni et al., 2022; Vu et al., 2020). Components of the liposomal lipid bilayer, lipoid S40 and CHOL, were first accurately weighed (Cubis® II Essentials MCE, Sartorius AG, Göttingen, Germany). The compositions of the liposomal formulas are listed in Table 1. Lipids were then dissolved in a system composed of chloroform and methanol in a ratio of 2:1 *v*/v, respectively. The organic solution was then transferred to a round-bottom flask and coupled to a BUCHI Rotavapor (Buchi R-215 Advanced Rotavapo with V-Condensor and V-850 Controller). The solvent was evaporated at 55 °C using a vacuum for one hour. This resulted in a thin film formation, which was kept under the same conditions for an additional 2 h to ensure full drying. Hydration of the film took place using double-distilled water for 2 h. Small unilamellar vesicles (SUV) were obtained using heat-controlled probe sonication (Fisher Bioblock Scientific S.A., Illkirch, France) at 750 W, 20 KHz, and 60 % amplitude for 5 min. SUV liposomes were then kept at 4 °C overnight.

**Table 1:** Compositions and physicochemical characteristics of the prepared plain and MLX-loaded liposomal systems.

Formula	Lip S40 (mg)	CHOL (mg)	MLX (mg)	P.S. (nm)	Span	EE (%)	DL (%)
**PL 1**	26	4	–	104.0 ± 2.00	1.55 ± 0.07	–	–
**PL 2**	26	2	–	116.7 ± 1.58	1.85 ± 0.05	–	–
**LMLX 1**	26	4	2	108.3 ± 1.53	1.32 ± 0.03	81.57 ± 0.57	4.62 ± 0.03
**LMLX 2**	26	2	1	108.0 ± 3.61	1.58 ± 0.09	87.08 ± 0.58	2.68 ± 0.02

### 2.3 Preparation of MLX-loaded liposomes

MLX-loaded SUV liposomes (LMLX 1 and LMLX 2) were prepared following the same procedures as plain liposomes, with the addition of MLX to the organic solution of the lipid phase.

### 2.4 Physicochemical characterization of MLX-loaded SUV liposomes

#### 2.4.1 Mean particle size and particle size distribution

The mean particle sizes of the produced formulations were measured using the dynamic laser light scattering method (Master Sizer 2000, version 5.54, Hydro 2000 S (Malvern Instruments Ltd., Worcestershire, UK) at room temperature (Vu et al., 2020). The distribution of particles in the population based on particle size was evaluated using the term “span,” a key indicator of particle population homogeneity.

#### 2.4.2 Entrapment efficiency and drug loading

The percentage of MLX entrapped inside the liposomal nanocarrier was evaluated using an indirect technique. Centrifugation of liposomal dispersion was done at 15,000 rpm, 4 °C, for 90 min (Vu et al., 2020) using a HERMLE Z 326 K centrifuge (HERMLE Labortechnik GmbH, Wehingen, Germany). The supernatant was decanted. The supernatant was then withdrawn and filtered through 0.2 μm nylon filter. The MLX concentration in the supernatant was detected using a UV–Vis spectrophotometer (Schimadzu UV–Vis spectrophotometer 1601, Schimadzu, Japan) at 359 nm (Yegireddy et al., 2022). Entrapment efficiency (EE)% in each sample was calculated using the following equation (Ahmed, 2020):

$$\mathrm{EE}\;\left(\%\right)=\frac{D_{T}-D_{F}}{D_{T}}$$

Where D_T_ is the total amount of the MLX originally integrated into the formula, and D_F_ is the estimated amount of free MLX in the supernatant. To ensure statistical validity, the experiment was replicated for each MLX-loaded SUV liposome formula.

Drug loading (DL) percentage was determined using the calculated amount of the free drug as EE%, but using the following equation:

$$\mathrm{DL}\;\left(\%\right)=\frac{D_{T}-D_{F}}{L_{T}\;}$$

Where L_T_ is the total amount of all the components of the liposomal nanocarrier (Akel et al., 2021; Liu et al., 2020).

#### 2.4.3 Physical stability study

The physical stability was studied by tracing the changes in particle size. Coalescence or aggregation of liposomal nanoparticles could be clearly manifested by increased particle size with frequent variations in the span or polydispersity index (PDI) values. Liposomal formulations were kept at 4 °C for 2 months. The particle size and span values of the formulas were measured after 1 and 2 months of preparation, respectively (Alomrani et al., 2019; Vu et al., 2020).

#### 2.4.4 Differential Scanning Calorimetry (DSC)

DSC was performed to detect any possible physical interaction(s) between the drug and any of the components of the liposomal nanocarrier. It was also useful in determining the state at which the drug occurs in the nanocarrier. A standard 40-μL aluminum pan was used to contain samples (1–3 mg each) of the pure MLX, liposome components, dried plain-formulated liposomes (PL 1), and selected dried MLX-loaded liposomes (LMLX 1). Each pan was sealed with a lid and crimped. Next, each pan was heated (rate of 10 °C/min., range of 25 °C to 350 °C) while being continuously purged of nitrogen (30 mL/min.) against a reference pan. Using a DSC calorimeter (Shimadzu DSC-60, Kyoto, Japan), variations in energy consumption were recorded, and thermograms were created (Li et al., 2021).

#### 2.4.5 Fourier transformation infrared (FT-IR) spectroscopy

The FT-IR spectra of MLX, dry PL 1, and dry LMLX 1 were analyzed using a Nicolet™ iS™ 5 FTIR Spectrometer (Thermo Fisher Scientific Inc., MA, USA) to identify any potential MLX-nano liposomal carrier interactions. Samples were mixed with potassium bromide (IR grade) in a 100:1 ratio and subjected to scanning within a wave number range of 4000 to 500 cm^−1^.

### 2.5 Morphological characterization

The LMLX 1 formula was situated on a copper grid with formvar film. It was stained using phosphotungstic acid (4 % *W*/W). The copper grid containing the stained sample was allowed to air dry at room temperature. The grid was securely fixed to the holder and inserted into the transmission electron microscope (TEM) (JEOL-2100, JEM-HR-2100, Tokyo, Japan) with an accelerated voltage of 200 kV and a beam current of 120 μA to examine the samples.

### 2.6 *In vitro* drug release

A comparative *in vitro* drug release investigation was conducted between LMLX 1 and the reference standard Mobitil® injection (MUP, Egypt) using the dialysis bag diffusion method. Mobitil® was chosen as a reference standard for *in vitro* release comparison because it has been widely used for the treatment of pain in the Egyptian market for more than 20 years. It has manifested great efficacy since then. A 5 mg aliquot of LMLX 1 formulation was put in a cellulose dialysis tube. Afterwards, the tightly sealed tube was placed in a vessel containing 100 mL of phosphate buffer at pH 8 (Akel et al., 2021; Yang et al., 2024). This dissolution medium was chosen to achieve sink condition for the release of MLX without affecting the differentiating and limiting capability of the test. The dissolution vessels were placed in a shaking water bath (Clifton, USA) at a temperature of 37 °C ± 0.5 and a 50-rpm shaking speed.

### 2.7 Mathematical modeling of *in vitro* release kinetics

The kinetics of MLX release from liposomal nanocarriers were calculated using the most commonly used release models, *i.e.*, zero-order, first-order, Higuchi, and Korsmeyer-Peppas models (Khorshid et al., 2022).

Graphs of the release kinetics models were created using the equations listed in Table 2, and linear regression for each system was calculated. The best-fitting model, *i.e.*, the model with the highest linear regression value, was considered the best model for describing the process of MLX release from the formulation.

**Table 2:** Mathematical modeling of MLX release kinetics from LMLX 1 as compared to Mobitil®.

Model	Equation⁎	Indication	Rate Constant / Correlation Coefficient	Mobitil®	LMLX 1
Zero-Order	$C=K_{0}t$	A constant rate of drug release	K_0_ R^2^	11.3700 0.5963	6.8476 0.8481
First-Order	$\mathrm{Log}\;\left(100-C\right)=-\frac{K_{f}t}{2.303}$	A concentration-dependent rate of drug release	K_f_ R^2^	0.6898 0.8713	0.0748 0.8973
Higuchi	$C=K_{H}\sqrt{t}$	Drug release is diffusion-dependent	K_H_ R^2^	32.4220 0.7422	28.6340 0.9677
Korsmeyer-Peppas	$C=K_{K}t^{n}$	Drug release is dissolution-dependent	n R^2^	0.2594 0.8255	0.5175 0.9606

⁎ *C* is the cumulative % of drug released at time t, K_0_ is the zero-order rate constant, K_f_ is the first-order rate constant, K_H_ is the Higuchi dissolution constant, K_K_ is the Korsmeyer-Peppas constant, and n is the exponent describing a specific diffusion process mechanism.

### 2.8 Pharmacokinetics, pharmacodynamics, and pain assessment studies in an *in vivo* pain model

A parallel prospective randomized experimental study was conducted alongside another experimental study on the application of 3D titanium miniplates for mandibular fracture in mongrel dogs (unpublished results) at the Department of Surgery, Anesthesiology, and Radiology, Faculty of Veterinary Medicine, Suez Canal University. The number of dogs was estimated after sample size calculation using G*Power version 3.1.9.2 (Faul et al., 2007). In accordance with previous studies (Farokhzad et al., 2021; Lafuente et al., 2005). The estimated sample size (*n*) = 12. Clinically healthy male mongrel dogs with an age range of 12–18 months and a weight of 12.5–17 kg were randomly allocated into two groups using the sealed white envelopes randomization procedure: the Mobitil® group (*n* = 6) and the LMLX 1 group (*n* = 6). The dogs were maintained on standard food according to NRC nutritional standards for dogs with free access to water (Council, 2006). The dogs were housed in the animal house for 14 days before the experiment to allow for acclimatization and a complete check for any health issues that may interfere with the study.

Prior to surgery, food and water were stopped for 12 and 2 h, respectively. The dogs were intramuscularly (IM) injected with acepromazine (Calmivet®, Vetoquinol, France) at a dosage of 0.05 mg/kg and 0.04 mg/kg atropine sulfate (Memphis Pharmaceutical, Egypt). MLX was given to both groups (Mobitil® and LMLX 1) at a dosage of 0.2 mg/kg. Induction of general anesthesia was done using an intravenous (IV) injection of 2.2 mg/kg Propofol (Diprivan®, AstraZeneca, UK) and maintained *via* 2 % isoflurane (IsoFlo®, Zoetis, USA) and oxygen (Clarke et al., 2014). During surgery, lactated Ringer's solution was administered IV at a rate of 10 mL/kg/h.

The same surgeon (M.A.H.) performed all the surgical procedures, including induction of the mandibular fracture and fixation using double square 3D titanium mini plates of 1.0 mm thickness fixed with 2.0 mm titanium screws of 7 mm length. Cefotaxime (50 mg/kg, PO, q 8 h) for 7 days was IM administered to all dogs. Mobitil® (0.2 mg/kg, PO, q 24 h) or the equivalent amount of LMLX 1 were given IM for three postoperative days. There were no additional NSAIDs, systemic corticosteroids, or opioids given postoperatively.

#### 2.8.1 Pharmacokinetics study

##### 2.8.1.1 Study design

The study was performed to detect the pharmacokinetic behavior of LMLX 1 in comparison to a reference standard MLX IM injection solution (Mobitil®). Mobitil® has been used for IM injection of meloxicam for both humans and animals for more than 20 years in the Egyptian market showing highly satisfactory results for pain relief. Blood samples were obtained from the cephalic vein before anesthesia induction (baseline) and at 0.25, 0.5, 1, 1.5, 2, 3, 4, 6, 8, 12, and 24 h after injection of the first bolus of MLX in both groups preoperatively.

##### 2.8.1.2 Quantification of drug in plasma

Plasma was separated for the analysis of the drug concentration. To precipitate proteins, 1 mL of acetonitrile was added to 1 mL of plasma. The mixture was centrifuged at 4500 rpm for 5 min at 4 °C. The supernatant was then filtered through a 0.2 μm nylon syringe filter into amber vials for analysis. The HPLC quantification method was performed using a 250 × 4.6 mm (i.d.) Phenomenex® (5-μm particle size) reversed-phase C18 analytical column (Phenomenex, Torrance, CA, USA). The mobile phase was prepared by mixing methanol and water at a ratio of 40:60 (*V*/V) and UV detection at 260 nm. The flow rate was 1 mL per minute. All measurements were done at 25 °C. The injection volume was 20 μL. Data collection was done using Class-VP software (Shimadzu, Kyoto, Japan.).

##### 2.8.1.3 Determination of pharmacokinetic behavior

The drug concentration-time curve was constructed for LMLX 1 and Mobitil® formulations. The determination of the pharmacokinetic behavior of the two MLX formulations was performed using five key parameters: C_max_, t_max_, t_1/2_, AUC_0__–__24_, and Cl/F. Data were analyzed using the pharmacokinetic PKsolver® 2.0 add-in for Microsoft Excel® software.

#### 2.8.2 Pharmacodynamics study

Blood specimens were collected in anticoagulant-free tubes before (baseline), and at 8, 16, 24, 32, 40, 48, 56, 64, 72, and 80 h post-operatively. The obtained sera were kept at −20 °C until biochemical analysis. Serum was utilized to investigate inflammatory status and alkaline phosphatase (ALP) activity. Interleukin-6 (IL-6) and C-reactive protein (CRP) were assayed using enzyme-linked immunosorbent assay (ELISA) kits following the labelled instructions (MyBioSource Co., Catalog No.: MBS453159 and MBS355410, respectively). Colorimetric kinetic determination of serum ALP activity was performed using a commercially available kit (BioAssay Systems, Catalog No.: DALP-250) following the manufacturer's protocol.

#### 2.8.3 Pain assessment study

Two blinded investigators to the treatment performed the pain assessment for the operated dogs. The pain assessment was conducted using two scoring systems: the short form of the Glasgow Composite Pain Scale (CMPS-SF) with 24 points (Reid et al., 2007) and the University of Melbourne pain scale (UMPS) with 27 points (Firth and Haldane, 1999; Hernández-Avalos et al., 2020). The assessments were done before anesthesia induction (baseline) and at specific time intervals after surgery (1, 2, 4, 6, 8, 12, 16, 20, 24, 36, 48, and 72 h). Rescue analgesia with Tramadol (Amriya for Pharmaceutical Industries, S.A.E.) was injected IV at a dose of 3 mg/kg when the CMPS-SF score was more than 6, considering the dog's clinical picture.

### 2.9 Statistical analysis

The normal distribution of the data was analyzed using the Kolmogorov-Smirnov normality test. The data were statistically described as mean ± standard deviation (SD). An independent sample *t*-test was used to compare the two groups. One-way ANOVA test was used to compare the time intervals in each group. Tukey's post-hoc tests were used for pairwise comparisons. For non-parametric variables, the Mann-Whitney test was used to compare the two groups. A *P* value <0.05 was considered to be statistically significant. All analysis was done using the computer program SPSS software for Windows version 26.0 (Statistical Package for Social Science, Armonk, NY: IBM Corp.). The graphs were created using GraphPad Prism software version 6 (GraphPad Software, Inc., USA).

## 3 Results and discussion

### 3.1 Evaluation of liposomes

#### 3.1.1 Particle size and span

Particle size is a key parameter that determines the intensity and efficiency of the nanocarrier system's characteristics. For instance, since dissolution is a surface phenomenon, as particle size decreases, the dissolution rate and even the intrinsic solubility of the drug increase. The average particle sizes and span values of the prepared liposomal formulations are listed in Table 1. All particles exhibited a size range of less than 120 nm. The size of all plain and MLX-loaded nanoparticles ranged from 104 to 116 nm. This size range was considered to be ideal for improving drug solubility and bioavailability (Batinić et al., 2020). By comparing the particle sizes of PL 1 and PL 2, it was evident that increasing the phospholipid ratio in the formula led to an increase in particle size. This might be illustrated by the fact that multilamellar vesicles are more likely to form at higher phospholipid loads, which has the effect of increasing the size of the nanoparticles produced. This was aligned with the findings of (Harbi et al., 2016) during the sertraline liposome development process.

It was also found that integrating MLX, a relatively hydrophobic drug with an aromatic structure, into the liposome resulted in a larger particle size. This might be attributed to the drug's hydrophobicity or chemical structure. MLX resided mostly in the lipid bilayer of liposomes, increasing its hydrophobicity and hence the particle size of the liposomes (Vu et al., 2020). Since MLX contains aromatic rings with some structural similarity to folic acid, the effect of its incorporation on the lipid membrane of liposomes resembled the effect of the latter observed by (Batinić et al., 2020). In their study, the incorporation of folic acid into liposomes increased particle size. They explained this action by pointing to folic acid's aromatic part, which contains a polar group. The polar group in the aromatic part of the phosphatidylcholine bilayer contributed to positioning the drug away from the inner portion of the bilayer and closer to the lipid-aqueous interface (Batinić et al., 2020).

**Fig. 1:**
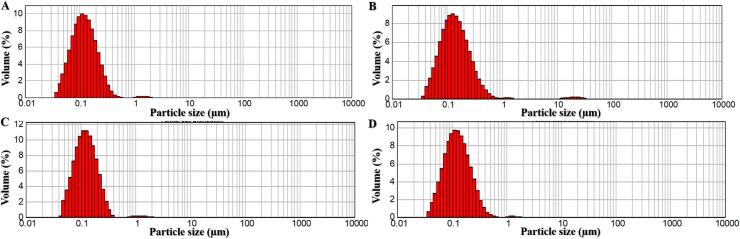
Particle size distribution curves of (a) PL 1, (b) PL 2, (c) LMLX 1, and (d) LMLX 2.

The particle size distribution of nanodrugs is a crucial characteristic that affects both safety and effectiveness and therefore has an impact on clinical findings (Rahman et al., 2018). The span value is a measure of the extent of the distribution of particle sizes. The increased magnitude of the span directly correlates with the width of particle sizes found in the powder (Moolchandani et al., 2015). All of the formulas created in this study had a span value of less than 1.8, suggesting a narrow particle size distribution. This showed the formulas were appropriate in terms of particle size distribution. Fig. 1 illustrates curves for particle size distributions of the prepared formulas.

#### 3.1.2 Entrapment efficiency and drug loading

This study revealed that the amount of MLX entrapped within the liposomes varied in response to the variation in liposomal composition. Increasing the drug/phospholipid ratio resulted in reducing EE. As shown in Table 1, EE% increased from 81.57 % to 87.08 % upon reducing the aforementioned ratio from 1:13 to 1:26, respectively. This effect was reported by (Ahmed, 2020), where the estimated effect of the drug/phospholipid molar ratio was negative, indicating a reverse proportionality. Furthermore, it has been proposed that this effect is increased by cholesterol levels (Shavi et al., 2016).

Meanwhile, DL decreased as a consequence of a lowered drug/phospholipid ratio (Table 1), with LMLX 1 loading 4.62 % and LMLX 2 loading 2.68 %. This might be attributed to the method by which DL was determined by comparing the amount of entrapped drug to the total weight of the carrier. Therefore, reducing the drug or phospholipid is expected to reduce the relative amount of the drug initially added, which leads to decreasing DL (Akel et al., 2021; Liu et al., 2020).

#### 3.1.3 Physical stability

**Fig. 2:**
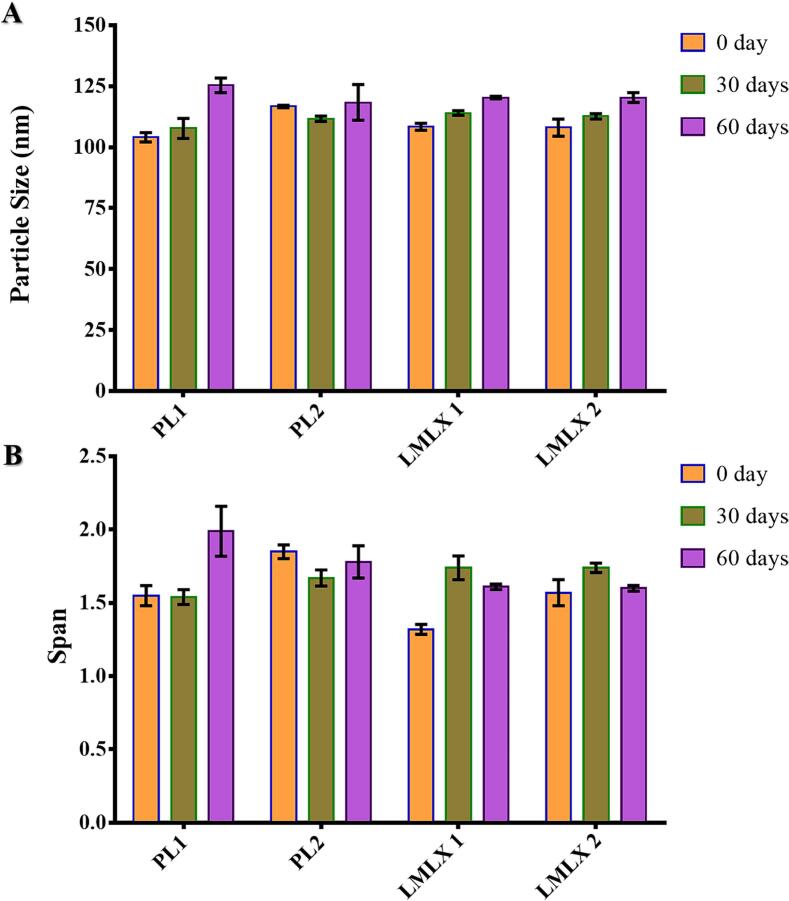
Liposomes stability as indicated by (A) particle size and (B) span at 0, 30, and 60 days of preparation.

Particle size and span value are basic criteria used to assess the stability of most lipid dispersions in aqueous media. This is because lipid dispersions are unstable, resulting in lipid coalescence or creaming (Pasarin et al., 2023). As demonstrated in Fig. 2, the produced liposomes showed a very slight increase in particle size (not more than 10 %) and a small rise in span values. This suggested that the liposomes were very stable throughout the entire study.

### 3.2 Selection of the optimum formula

The optimal formulation for IM injection into the dogs was chosen for further testing based on the properties of the optimum IM drug delivery system. The characteristics of LMLX 1 and LMLX 2 were comparable; nevertheless, a variation in the key variable of drug loading resulted in the selection of LMLX 1 as the optimal delivery system. This might be rationalized based on the restricted amount available for IM injection per single location (Orpet, 2018).

### 3.3 Physicochemical characterization of optimum formula

#### 3.3.1 Differential scanning calorimetry

**Fig. 3:**
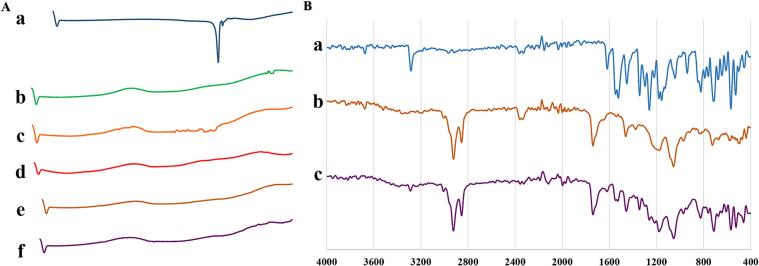
(A) DSC thermograms of (a) pure meloxicam, (b) Lipoid S 40, (c) Pure CHOL, (d) physical mixture of liposomal components, (e) PL 1, and (f) LMLX 1. (B) FT-IR spectroscopy of (a) pure meloxicam, (b) PL 1, and (c) LMLX 1.

The DSC thermogram (Fig. 3A) of pure MLX showed a sharp endothermic peak at 263 °C, representing the melting process followed by MLX breakdown, as exhibited by a small, broad peak immediately after the melting peak (Alshaikh et al., 2019). The main peak vanished upon simple mixing of the drug with liposome components or upon loading the drug into liposomes. This suggested that MLX dissolved completely in phospholipid (Li et al., 2021). The decomposition peak was shifted toward a higher temperature, indicating MLX stabilization upon incorporation into liposomes.

#### 3.3.2 FT-IR spectroscopy

The FT-IR spectrum of MLX showed prominent absorption bands at different frequencies (Fig. 3B). The frequency at which each characteristic band was depicted and the interpretation of these peaks are listed in Table 3. The FT-IR spectrum of LMLX 1 depicted the same specific peaks as in the pure MLX spectrum, with minor changes. This evidenced that the chemical structure of MLX remained unchanged. However, broadening, overlapping, weakening, and/or disappearance of some bands in the spectra of the LMLX 1 formulation were observed. This confirmed the effective encapsulation of MLX in the prepared nanovesicles (Ahmed, 2020).

**Table 3:** FT-IR spectrum band assignments of meloxicam.

Frequency (cm^−1^)	Intensity⁎	Characteristic of
3282	S	Stretching Vibrations of (N—H) _amide_
1614	S	Stretching Vibrations of (C=O) _amide_
1550	S	Stretching Vibrations of (C=N) _thiazol ring_
1342	S	Stretching Vibrations of (SO2) _asymmetric_
1182	S	Stretching Vibrations of (SO2) _symmetric_

⁎ S = Strong.

#### 3.3.3 Morphological characterization

**Fig. 4:**
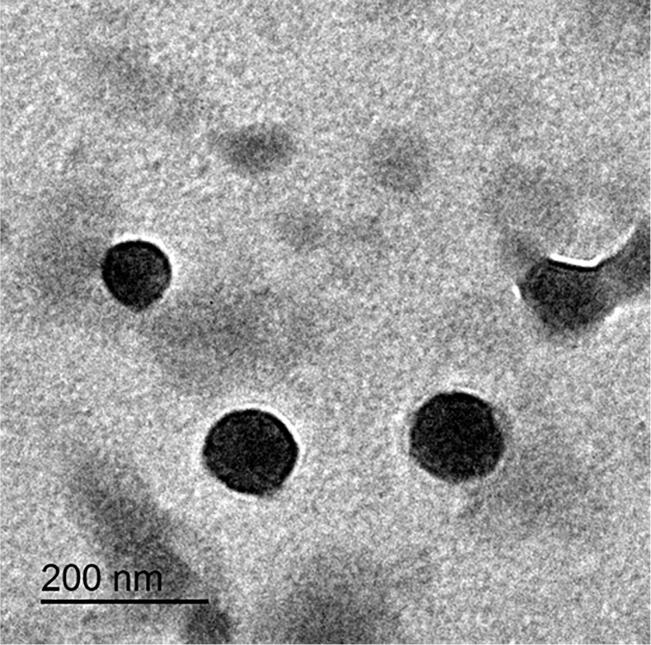
Transmission electron micrograph of LMLX 1.

The vesicular nature of LMLX 1 was verified by examining its morphological characteristics using TEM (Fig. 4). The selected formula LMLX 1 exhibited that particles were unilamellar and spherical.

#### 3.3.4 *In vitro* drug release

**Fig. 5:**
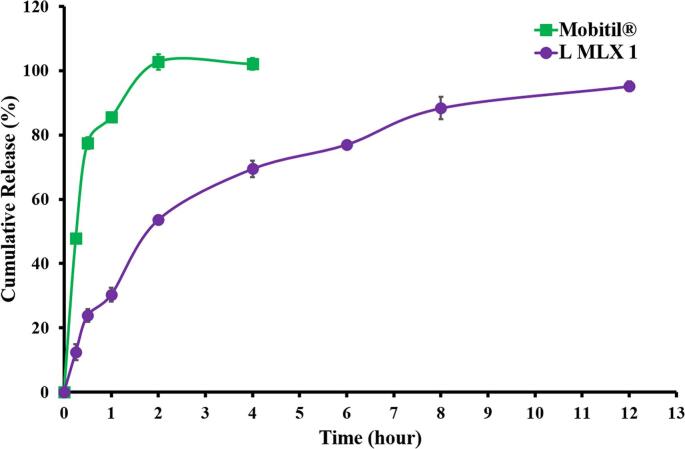
Release patterns of the selected formula (LMLX 1) in comparison to the reference standard, Mobitil®.

The release profile of MLX from Mobitil® was demonstrated to be rapid (Fig. 5). Almost 100 % of the amount was released after 2 h. This was attributed to the fact that MLX was formulated as a solution for injection with the aid of propylene glycol in the product of Mobitil® IM injection. On the other hand, MLX release from the liposomal nanocarrier was characterized by a slower rate as compared to Mobitil®. This was clarified by the method used to release lipophilic drugs from lipoidal nanocarriers, as demonstrated by the release kinetics.

The controlled release of MLX from the drug nanocarrier is preferred in many cases since the MLX half-life is relatively short (Yang et al., 2024). MLX is one of the NSAIDs that is used for treating pain, so the MLX formulation, as liposome-loaded, optimized its release in a controlled manner that is appropriate for the desired clinical effect in pain treatment, *i.e.*, slow enough to make a sustained effect and yet not too slow to make a prompt pain-relieving effect (Baek et al., 2017). This was evidenced by the release curve, where almost 25 % of the loaded amount was released during the first half hour while maintaining the controlled release of 65 % of the loaded amount during the following 7 h.

The control of drug release from a dosage form may involve one or more of the following phenomena, to name a few: drug type(s), incorporated drug dose(s), types and amounts of excipients, preparation technique, environmental conditions during drug release, as well as the geometry and dimensions of the drug delivery system (Siepmann and Siepmann, 2008). MLX is considered a small molecule with a molecular weight of 351.4 g/ mol (Luger et al., 1996). Small molecules can pass easily through the liposomal membranes. However, the addition of CHOL to the components of the liposomal nanocarrier resulted in increasing the rigidity of the membrane and its resistance to passage of the molecules through it (Haghiralsadat et al., 2018; Mehanna et al., 2009; Nie et al., 2012; Wu et al., 2019).

#### 3.3.5 Mathematical modeling of *in vitro* release kinetics

Four models for studying the release kinetics of MLX from LMLX 1 and the Mobitil® reference standard were utilized. Each of these four models reflects a mechanism by which the drug was released (Table 2).

As presented in Table 2, the Higuchi model exhibited the highest correlation coefficient (R^2^) for LMLX 1, followed by Korsemeyer-Peppas. This indicated that the primary mechanism involved in MLX release from the liposomes was the diffusion mechanism. This was attributed to the drug's lipophilic nature and its ability to diffuse through a lipoidal nanocarrier membrane. (Vu et al., 2020). Although the Higuchi model is not commonly employed for drug release from liposomal nanocarriers, numerous scientists have observed that the drug release from liposomal nanocarriers adheres to this model (Haeri et al., 2011; Jain and Jain, 2016; Mehanna et al., 2009; Vali et al., 2008; Vu et al., 2020). Many other studies reported that the drug release from the liposomal nanocarrier followed the Korsemeyer-Peppas model (Fernández-Romero et al., 2018; Jeon et al., 2015; Wu et al., 2019). The Korsemeyer-Peppas model is usually characterized by biphasic release, where there is a burst release of the adsorbed on the surface of liposomes or unentrapped in liposomes (Fernández-Romero et al., 2018; Lapenda et al., 2013; Wang et al., 2016). For a drug that is released by Higuchi or the Korsmeyer-Peppas model, it is suggested that its release takes place through diffusion (Fernández-Romero et al., 2018; Mehanna et al., 2009; Vu et al., 2020; Wu et al., 2019). Studying the regression coefficient (R^2^) of the Higuchi and Korsmeyer-Peppas models on the same formulations revealed that some formulations exhibit a very narrow difference between the two regression coefficients (Jeon et al., 2015; Mehanna et al., 2009; Vu et al., 2020). The Higuchi model is used when there is good control over the drug release, while the Korsmeyer-Peppas model works when there is a burst release followed by a controlled release of the drug. It has been reported that as the component that retards drug release in the liposomal formulation grows in concentration, the release kinetics gradually shift from the Korsmeyer-Peppas model to the Higuchi model. This was indicated by the values of the R^2^ of both models regarding the same formulation. This was reported when cholesterol concentration increased (Mehanna et al., 2009) and when the number of layers coating the liposomes increased (Jeon et al., 2015).

In terms of liposomal formulation in this study, the Higuchi model R^2^ dominated, while the Korsmeyer-Peppas model was very close to the former. This indicated that there was no prominent burst release and that the drug entrapment efficiency was relatively high. The mechanism attributed to the drug release was suggested to be the diffusion of MLX through the liposomal membrane to the release medium (Vu et al., 2020).

For Mobitil®, it was found that first-order kinetics was the best fit for drug release, showing that, being soluble, MLX release from the Mobitil® formulation depended on the remaining concentration of the drug.

### 3.4 The pharmacokinetics study

**Fig. 6:**
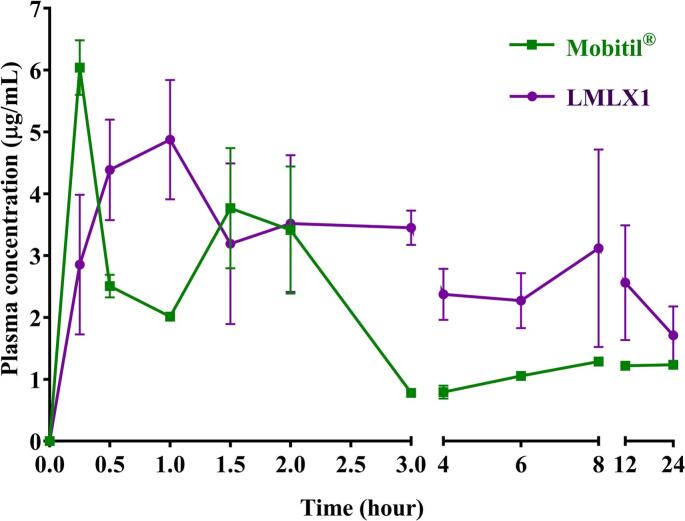
The drug plasma concentration-time profile after IM administration of LMLX 1 in comparison to the reference standard, Mobitil®.

Following IM administration of both Mobitil® and liposomal MLX formulation (LMLX 1), separately, a great difference in pharmacokinetic behavior was found (Fig. 6). The selected key pharmacokinetic parameters of both formulations were listed in Table 4. A soluble MLX formulation (Mobitil®^)^ showed a higher C_max_ with a lower t_max_ (6.04 μg/mL and 0.25 h, respectively) as compared to the LMLX 1 formulation (C_max_ = 4.88 and t_max_ = 1 h). However, the difference in maximum drug concentration in plasma between the two formulations was shown to be statistically non-significant at *P* < 0.05. This suggested that the only advantage of using soluble MLX over the liposomal formulation was the t_max_. However, knowing that the minimum plasma effective concentration of MLX was reported to be 0.7 μg/mL, the privilege of a lower t_max_ of Mobitil® was diminished (Wani et al., 2013). On the other hand, the liposomal formulation of MLX has resulted in a higher AUC_0__–__24_ and t_1/2_ combined with lower Cl/F suggesting a longer duration of action and a higher therapeutic outcome (Scheff et al., 2011).

**Table 4:** Pharmacokinetic parameters of LMLX 1 as compared to Mobitil® after IM injection in dogs.

Parameter	Mobitil®	LMLX 1
C_max_ (μg/mL)	6.04 ± 1.43	4.88 ± 0.91
t_max_ (h) ⁎	0.25 ± 0.11	1 ± 0.36
t_1/2_ (h) ⁎	16.53 ± 1.04	18.90 ± 1.27
Cl/F (mg)/(μg/mL)/h) ⁎	0.04 ± 0.01	0.02 ± 0.008
AUC_0__–__24_ (μg/mL⁎h) ⁎	33.00 ± 9.44	61.28 ± 12.63

⁎ Denotes significant difference between LMLX 1 and Mobitil® values at significance level (*P* < 0.05).

Furthermore, it was also shown that LMLX 1 manifested less fluctuating concentrations as compared to Mobitil® (Fig. 6), suggesting a more consistent drug concentration, fewer side effects, and a more persistent effect of the drug (Scheff et al., 2011).

### 3.5 The pharmacodynamics study

Fractures cause a series of events that can result in effective repair and function restoration or significant consequences, depending on intrinsic and extrinsic factors. The inflammatory reaction at the injury site is the first of four steps required for the healing process (Li et al., 2019). Bone injury triggers the release of pro-inflammatory cytokines, including TNF-α and interleukins (IL-1, IL-6, IL-8, IL-11, and IL-23) that induce an immunological response (Maruyama et al., 2020). Furthermore, IL-6 is considered a key stimulus that controls all proteins involved in the acute-phase response (Tanaka et al., 2014).

**Fig. 7:**
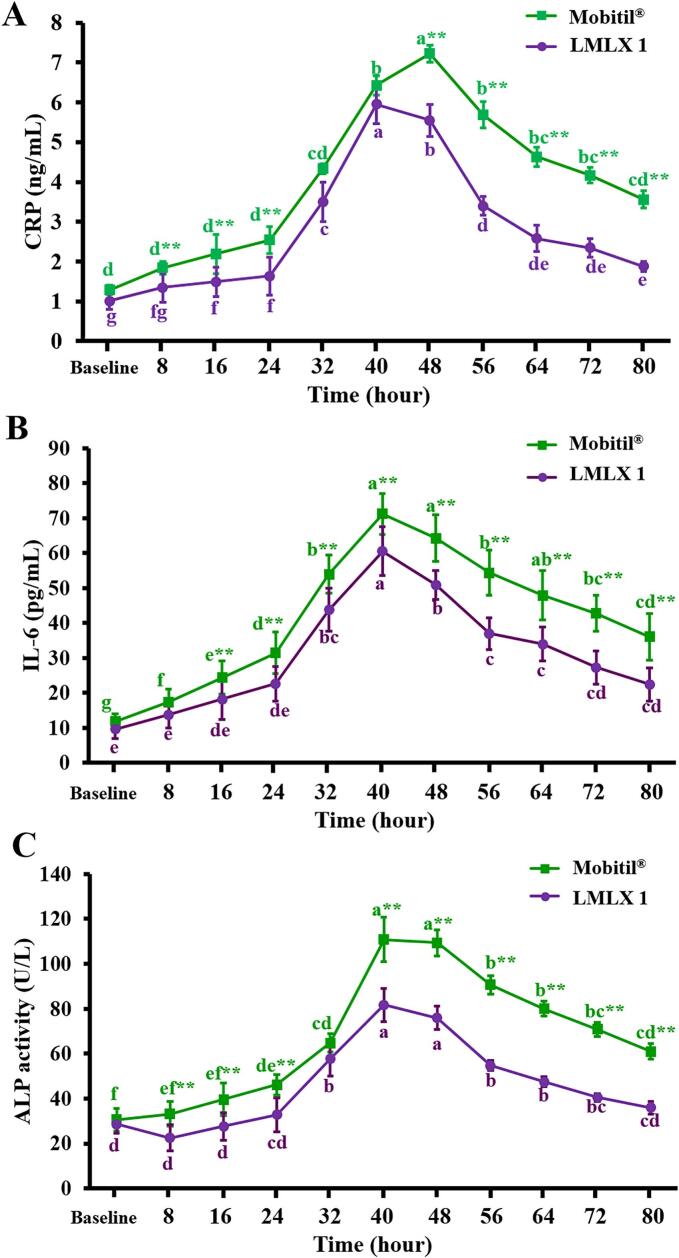
Effect of LMLX 1 *vs.* Mobitil® treatment on serum levels of (A) CRP, (B) IL-6, and (C) ALP. Data were represented as mean ± SD. Within the same group, different letters indicate a significant difference (*P* < 0.05). At the same time point, * indicates a significant difference (*P* < 0.05), and ** indicates a significant difference (*P* < 0.01) between the two groups.

CRP is an important acute-phase protein (APP) that is produced by hepatocytes in significant amounts following the induction of IL-6. As a non-specific APP, CRP is valuable for monitoring the body's response to various treatments (Raj et al., 2015). Furthermore, CRP has been correlated with both short- and long-term clinical outcomes (Zeng et al., 2021). In the present study, IL-6 and CRP levels increased significantly in both groups following mandible fracture (Fig. 7 A&B), indicating the incidence of an inflammatory response. These results were consistent with other reports that have found these inflammatory biomarkers elevated during bone injuries (Pape et al., 2007; Tylicka et al., 2021; Yang et al., 2007). The released inflammatory mediators have been linked with pain sensitization (Simon et al., 2023). Early intervention in controlling inflammation has an advantageous effect on the process of fracture healing. Conversely, strong and long-lasting inflammation could have detrimental effects (Chen et al., 2020; O'Keefe and Mao, 2011). NSAIDs are one of the main pharmacological groups used to manage post-traumatic inflammation and associated pain (Ghlichloo and Gerriets, 2023). The present findings demonstrated that using MLX in both groups resulted in a considerable successive drop in serum levels of IL-6 and CRP with time starting from 48 and 56 h, respectively, to the end of the experiment. Moreover, LMLX1 showed effective anti-inflammatory potential compared to Mobitil®, as indicated by lower mean values at different time points. It alleviates the body's response to the fracture by inducing better control of inflammatory mediators at most times.

ALP is a membrane-bound enzyme that catalyzes the hydrolysis of organic phosphate esters. It is one of the main markers of bone metabolism. Its activity in the serum rises in association with hepatobiliary and bone diseases (Song, 2017). Our data demonstrated that fracture significantly increased serum ALP activity in both groups as time increased to 40 h (Fig. 7C). However, when comparing the effects of both drugs, LMLX 1 exhibited the advantage of triggering decreased ALP activity in most periods, indicating that it could result in improved bone healing.

### 3.6 The outcomes of the pain assessment scales in the pain model

**Fig. 8:**
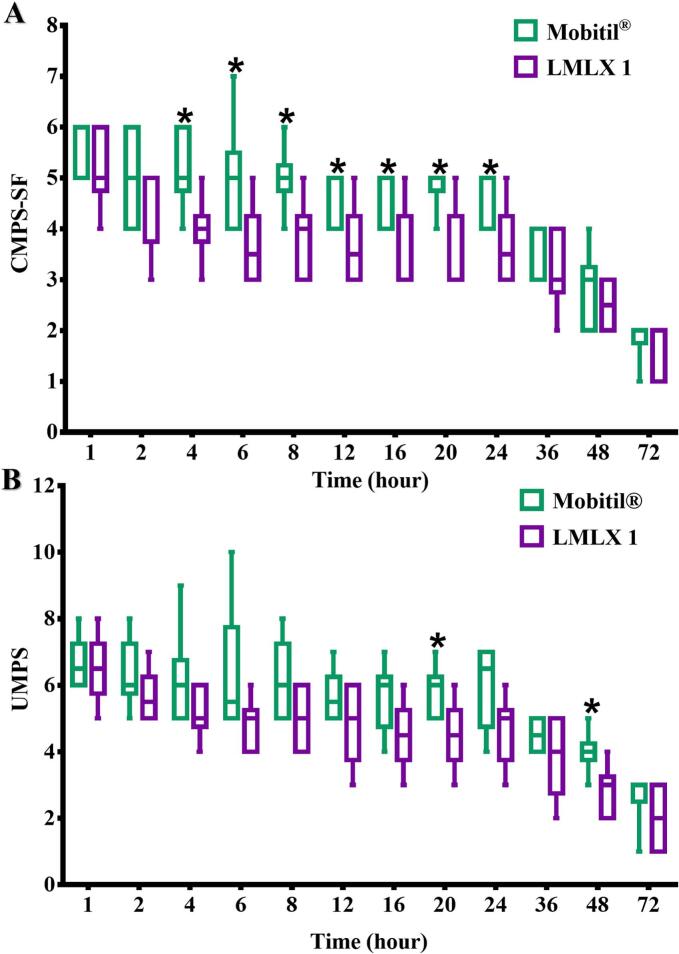
Box-and-whisker graphs show (A) CMPS-SF and (B) UMPS scores at different postoperative periods. The box represents the 25th and 75th percentile of data, with the horizontal line within indicating the median. The upper and lower whiskers indicate the range of values. The lack of a visible median line indicates the median value is toward the bottom of the box. The lack of a whisker indicates the absence of data within the relevant distance from the median, respectively. * denotes significance *P* < 0.05 between the two groups at the same time point.

The results of the CMPS-SF and UMPS scales revealed no evidence of substantial postoperative pain in either group. Regardless of the scoring system employed, the scores for measuring postoperative pain were found to be lower than the planned rescue levels in all of the groups studied. However, one dog in the Mobitil® group surpassed the rescue point (6) on the CMPS-SF scale and received analgesic treatment 6 h after surgery. Throughout the postoperative period, both study groups showed a decrease in pain scores on both scales (Fig. 8). During the monitoring period, no adverse effects were recorded in both groups related to the drug used.

### 3.7 Limitations and future perspectives

The possible limitation of this study is that the method for preparing liposomal nanocarriers is difficult to scale up on an industrial scale. Furthermore, the liposomal MLX IM injections' side effects were not determined. Further work is required to detect the safety, toxicity, and adverse effects of IM injection of liposomal nanocarriers loaded with MLX. Studies of the histopathological changes at the injection site (immediately and after chronic administration of the nanocarriers) should be carried out to transfer this technology to an industrial scale for obtaining a drug that could be marketed worldwide.

## 4 Conclusion

The incorporation of MLX into liposomes was evidenced to modify many characteristics of the MLX *in vitro* and *in vivo*. The prepared MLX-loaded liposomes manifested a well-acknowledged particle size (108 nm) with an almost monodispersed population (span = 1.32). The LMLX 1 formula showed high entrapment of the drug (81.57 %) with an appropriate loading percentage (4.62 %), hence its appropriate applicability for IM injection. The formulation exhibited high stability with no drastic chemical or physical interaction between the drug and the carrier, as illustrated by the stability study, DSC, and FT-IR spectroscopy. Incorporated into a liposomal formulation, MLX showed controlled *in vitro* release through a diffusion mechanism. Upon correlation with *in vitro* release, an *in vivo* pharmacokinetics study revealed a controlled release of the drug with an almost steady drug concentration in the blood, allowing less side effects and a high therapeutic outcome as compared to Mobitil®. The findings of the *in vivo* pharmacodynamics confirmed the superior behavior of LMLX 1 over Mobitil®, as all the pain markers showed a significant decrease over time between the two formulas. To sum up, the use of preoperative and postoperative liposomal MLX administration for three consecutive days proved to be a safe and effective approach to managing postoperative pain in dogs undergoing orthopedic surgery.

## Ethical approval statement

The Faculty of Veterinary Medicine's Institutional Animal Use and Care Committee examined and approved all experimental protocols (Approval Number 2022015) in compliance with the ARRIVE guidelines.

## Funding

This research did not receive any specific grant from funding agencies in the public, commercial, or not-for-profit sectors.

## CRediT authorship contribution statement

**Pierre A. Hanna:** Writing – review & editing, Writing – original draft, Validation, Supervision, Methodology, Investigation, Formal analysis, Data curation, Conceptualization. **Hatim A. Al-Abbadi:** Writing – original draft, Resources, Formal analysis, Conceptualization. **Mohamed A. Hashem:** Writing – original draft, Methodology, Formal analysis, Data curation, Conceptualization. **Aziza E. Mostafa:** Writing – original draft, Validation, Methodology, Formal analysis, Data curation. **Yasmina K. Mahmoud:** Writing – original draft, Validation, Methodology, Formal analysis, Data curation. **Eman A. Ahmed:** Writing – original draft, Methodology, Formal analysis, Data curation. **Ibrahim E. Helal:** Writing – original draft, Validation, Resources, Investigation, Formal analysis, Data curation. **Mahmoud F. Ahmed:** Writing – review & editing, Visualization, Validation, Supervision, Investigation, Formal analysis, Data curation, Conceptualization.

## Declaration of competing interest

The authors declare that they have no known competing financial interests or personal relationships that could have appeared to influence the work reported in this paper.

## Data Availability

The data that support the findings of this study are available from the corresponding authors, Pierre A. Hanna and Mahmoud F. Ahmed upon reasonable request.

## References

[bb0005] Ahmed T.A. (2020). Development of rosuvastatin flexible lipid-based nanoparticles: promising nanocarriers for improving intestinal cells cytotoxicity. BMC Pharmacol. Toxicol..

[bb0010] Akel H., Ismail R., Katona G., Sabir F., Ambrus R., Csóka I. (2021). A comparison study of lipid and polymeric nanoparticles in the nasal delivery of meloxicam: Formulation, characterization, and in vitro evaluation. Int. J. Pharm..

[bb0015] Alomrani A., Badran M., Harisa G.I., Alhariri M., Alshamsan A., Alkholief M. (2019). The use of chitosan-coated flexible liposomes as a remarkable carrier to enhance the antitumor efficacy of 5-fluorouracil against colorectal cancer. Saudi Pharm J.

[bb0020] Alshaikh R.A., Essa E.A., El Maghraby G.M. (2019). Eutexia for enhanced dissolution rate and anti-inflammatory activity of nonsteroidal anti-inflammatory agents: Caffeine as a melting point modulator. Int. J. Pharm..

[bb0025] Baek J.S., Yeo E.W., Lee Y.H., Tan N.S., Loo S.C.J. (2017). Controlled-release nanoencapsulating microcapsules to combat inflammatory diseases. Drug Des. Devel. Ther..

[bb0030] Batinić P.M., Đorđević V.B., Stevanović S.I., Balanč B.D., Marković S.B., Luković N.D., Mijin D.Ž., Bugarski B.M. (2020). Formulation and characterization of novel liposomes containing histidine for encapsulation of a poorly soluble vitamin. J. Drug Deliv. Sci. Technol..

[bb0035] Bhalani D.V., Nutan B., Kumar A., Singh Chandel A.K. (2022). Bioavailability enhancement techniques for poorly aqueous soluble drugs and therapeutics. Biomedicines.

[bb0040] Chen Z., Xie L., Xu J., Lin X., Ye J., Shao R., Yao X. (2020). Changes in alkaline phosphatase, calcium, C-reactive protein, D-dimer, phosphorus and hemoglobin in elderly osteoporotic hip fracture patients. Ann. Palliat. Med..

[bb0045] Clarke, K.W., Trim, C.M., Hall, L.W., 2014. Anaesthesia of the dog, in: Clarke, K.W., Trim, C.M., Hall, L.W. (Eds.), Veterinary Anaesthesia 11th ed. W.B. Saunders, Oxford, pp. 405–498.

[bb0050] Council N.R. (2006).

[bb0055] Deneuche A.J., Dufayet C., Goby L., Fayolle P., Desbois C. (2004). Analgesic comparison of meloxicam or ketoprofen for orthopedic surgery in dogs. Vet. Surg..

[bb0060] Farokhzad B., Sabiza S., Razi Jalali M., Baniadam A. (2021). Intraperitoneal administration of lidocaine or tramadol alone or in combination on postoperative pain after ovariohysterectomy in dogs. Vet. Med. Sci..

[bb0065] Faul F., Erdfelder E., Lang A.G., Buchner A. (2007). G*Power 3: a flexible statistical power analysis program for the social, behavioral, and biomedical sciences. Behav. Res. Methods.

[bb0070] Fernández-Romero A.M., Maestrelli F., Mura P.A., Rabasco A.M., González-Rodríguez M.L. (2018). Novel findings about double-loaded curcumin-in-HPβcyclodextrin-in liposomes: effects on the lipid bilayer and drug release. Pharmaceutics.

[bb0075] Firth A.M., Haldane S.L. (1999). Development of a scale to evaluate postoperative pain in dogs. J. Am. Vet. Med. Assoc..

[bb0080] Ghlichloo I., Gerriets V. (2023). StatPearls Publishing Copyright © 2023.

[bb0085] Glavas-Dodov M., Fredro-Kumbaradzi E., Goracinova K., Simonoska M., Calis S., Trajkovic-Jolevska S., Hincal A.A. (2005). The effects of lyophilization on the stability of liposomes containing 5-FU. Int. J. Pharm..

[bb0090] Gruet P., Seewald W., King J.N. (2011). Evaluation of subcutaneous and oral administration of robenacoxib and meloxicam for the treatment of acute pain and inflammation associated with orthopedic surgery in dogs. Am. J. Vet. Res..

[bb0095] Haeri A., Sadeghian S., Rabbani S., Anvari M.S., Boroumand M.A., Dadashzadeh S. (2011). Use of remote film loading methodology to entrap sirolimus into liposomes: preparation, characterization and in vivo efficacy for treatment of restenosis. Int. J. Pharm..

[bb0100] Haghiralsadat F., Amoabediny G., Helder M.N., Naderinezhad S., Sheikhha M.H., Forouzanfar T., Zandieh-Doulabi B. (2018). A comprehensive mathematical model of drug release kinetics from nano-liposomes, derived from optimization studies of cationic PEGylated liposomal doxorubicin formulations for drug-gene delivery. Artif. Cells Nanomed. Biotechnol..

[bb0105] Harbi I., Aljaeid B., El-Say K.M., Zidan A.S. (2016). Glycosylated sertraline-loaded liposomes for brain targeting: QbD study of formulation variabilities and brain transport. AAPS PharmSciTech.

[bb0110] Hernández-Avalos I., Valverde A., Ibancovichi-Camarillo J.A., Sánchez-Aparicio P., Recillas-Morales S., Osorio-Avalos J., Rodríguez-Velázquez D., Miranda-Cortés A.E. (2020). Clinical evaluation of postoperative analgesia, cardiorespiratory parameters and changes in liver and renal function tests of paracetamol compared to meloxicam and carprofen in dogs undergoing ovariohysterectomy. PLoS One.

[bb0115] Jain A., Jain S.K. (2016). In vitro release kinetics model fitting of liposomes: an insight. Chem. Phys. Lipids.

[bb0120] Jeon S., Yoo C.Y., Park S.N. (2015). Improved stability and skin permeability of sodium hyaluronate-chitosan multilayered liposomes by layer-by-layer electrostatic deposition for quercetin delivery. Colloids Surf. B: Biointerfaces.

[bb0125] Khalil N.Y., Aldosari K.F., Brittain H.G. (2020). Profiles of Drug Substances, Excipients and Related Methodology.

[bb0130] Khorshid S., Montanari M., Benedetti S., Moroni S., Aluigi A., Canonico B., Papa S., Tiboni M., Casettari L. (2022). A microfluidic approach to fabricate sucrose decorated liposomes with increased uptake in breast cancer cells. Eur. J. Pharm. Biopharm..

[bb0135] Kumari L., Choudhari Y., Patel P., Gupta G.D., Singh D., Rosenholm J.M., Bansal K.K., Kurmi B.D. (2023). Advancement in solubilization approaches: a step towards bioavailability enhancement of poorly soluble drugs. Life (Basel).

[bb0140] Kumbham S., Ajjarapu S., Ghosh B., Biswas S. (2023). Current trends in the development of liposomes for chemotherapeutic drug delivery. J. Drug Deliv. Sci. Technol..

[bb0145] Kwok P.C., Chan H.K. (2014). Nanotechnology versus other techniques in improving drug dissolution. Curr. Pharm. Des..

[bb0150] Lafuente M.P., Franch J., Durall I., Díaz-Bertrana M.C., Márquez R.M. (2005). Comparison between meloxicam and transdermally administered fentanyl for treatment of postoperative pain in dogs undergoing osteotomy of the tibia and fibula and placement of a uniplanar external distraction device. J. Am. Vet. Med. Assoc..

[bb0155] Lapenda T.L., Morais W.A., Almeida F.J., Ferraz M.S., Lira M.C., Santos N.P., Maciel M.A., Santos-Magalhães N.S. (2013). Encapsulation of trans-dehydrocrotonin in liposomes: an enhancement of the antitumor activity. J. Biomed. Nanotechnol..

[bb0160] Lee M.K. (2020). Vivo Evidence and Recent Approaches. Pharmaceutics 12.

[bb0165] Li J., Kacena M.A., Stocum D.L., Burr D.B., Allen M.R. (2019). Basic and Applied Bone Biology.

[bb0170] Li J., Chang C., Zhai J., Yang Y., Yu H. (2021). Ascorbyl palmitate effects on the stability of curcumin-loaded soybean phosphatidylcholine liposomes. Food Biosci..

[bb0175] Liu Y., Yang G., Jin S., Xu L., Zhao C.X. (2020). Development of high-drug-loading nanoparticles. Chempluschem.

[bb0180] Luger P., Daneck K., Engel W., Trummlitz G., Wagner K. (1996). Structure and physicochemical properties of meloxicam, a new NSAID. Eur. J. Pharm. Sci..

[bb0185] Maruyama M., Rhee C., Utsunomiya T., Zhang N., Ueno M., Yao Z., Goodman S.B. (2020). Modulation of the inflammatory response and bone healing. Front. Endocrinol. (Lausanne).

[bb0190] Mehanna M.M., Elmaradny H.A., Samaha M.W. (2009). Ciprofloxacin liposomes as vesicular reservoirs for ocular delivery: formulation, optimization, and in vitro characterization. Drug Dev. Ind. Pharm..

[bb0195] Moolchandani V., Augsburger L.L., Gupta A., Khan M., Langridge J., Hoag S.W. (2015). Characterization and selection of suitable grades of lactose as functional fillers for capsule filling: part 1. Drug Dev. Ind. Pharm..

[bb0200] Moraes É.B., Antunes J.M., Ferrari M.F.M., Fontes B.V., Pereira R., Ogawa L., Daher D.V. (2024). Post-operative pain management by nurses in an intensive care unit: a best practice implementation project. JBI Evid. Implement..

[bb0205] Mwema A., Muccioli G.G., Des Rieux A. (2023). Innovative drug delivery strategies to the CNS for the treatment of multiple sclerosis. J. Control. Release.

[bb0210] Ni Y., Zhao W., Cheng W., Deng C., Ying Z., Li L., Wang X., Sun C., Tu J., Jiang L. (2022). Lipopeptide liposomes-loaded hydrogel for multistage transdermal chemotherapy of melanoma. J. Control. Release.

[bb0215] Nie Y., Ji L., Ding H., Xie L., Li L., He B., Wu Y., Gu Z. (2012). Cholesterol derivatives based charged liposomes for doxorubicin delivery: preparation, in vitro and in vivo characterization. Theranostics.

[bb0220] O’Keefe R.J., Mao J. (2011). Bone tissue engineering and regeneration: from discovery to the clinic--an overview. Tissue Eng. Part B Rev..

[bb0225] Orpet H., Coombes N., Silva-Fletcher A. (2018). Veterinary Clinical Skills Manual.

[bb0230] Pape H.-C., Tsukamoto T., Kobbe P., Tarkin I., Katsoulis S., Peitzman A. (2007). Assessment of the clinical course with inflammatory parameters. Injury.

[bb0235] Parekh V.J., Desai N.D., Shaikh M.S., Shinde U.A. (2017). Self nanoemulsifying granules (SNEGs) of meloxicam: preparation, characterization, molecular modeling and evaluation of in vivo anti-inflammatory activity. Drug Dev. Ind. Pharm..

[bb0240] Pasarin D., Ghizdareanu A.I., Enascuta C.E., Matei C.B., Bilbie C., Paraschiv-Palada L., Veres P.A. (2023). Coating materials to increase the stability of liposomes. Polymers (Basel).

[bb0245] Paul S., Strelchik A., O’Day J., Guedes A.G.P., Gordon-Evans W.J. (2024). Comparison of bupivacaine liposome injectable solution and fentanyl for postoperative analgesia in dogs undergoing limb amputation. Vet. Surg..

[bb0250] Petrović S., Tačić A., Savić S., Nikolić V., Nikolić L., Savić S. (2017). Sulfanilamide in solution and liposome vesicles; in vitro release and UV-stability studies. Saudi Pharm J.

[bb0255] Rahman Z., Charoo N.A., Akhter S., Beg S., Reddy I.K., Khan M.A., Grumezescu A.M. (2018). Nanoscale Fabrication, Optimization, Scale-Up and Biological Aspects of Pharmaceutical Nanotechnology.

[bb0260] Raj D.S., Pecoits-Filho R., Kimmel P.L., Kimmel P.L., Rosenberg M.E. (2015). Chronic Renal Disease.

[bb0265] Reid J., Nolan A., Hughes J., Lascelles D., Pawson P., Scott E. (2007). Development of the short-form Glasgow Composite Measure Pain Scale (CMPS-SF) and derivation of an analgesic intervention score. Animal Welfare-Potters Bar Then Wheathampstead.

[bb0270] Scheff J.D., Almon R.R., Dubois D.C., Jusko W.J., Androulakis I.P. (2011). Assessment of pharmacologic area under the curve when baselines are variable. Pharm. Res..

[bb0275] Shah S., Mori D., Prajapati B., Vyas A.J., Soniwala M., Prajapati B., Patel J. (2023). Lipid-Based Drug Delivery Systems: Principles and Applications.

[bb0280] Shavi G.V., Sreenivasa Reddy M., Raghavendra R., Nayak U.Y., Kumar A.R., Deshpande P.B., Udupa N., Behl G., Dave V., Kushwaha K. (2016). PEGylated liposomes of anastrozole for long-term treatment of breast cancer: in vitro and in vivo evaluation. J. Liposome Res..

[bb0285] Siepmann J., Siepmann F. (2008). Mathematical modeling of drug delivery. Int. J. Pharm..

[bb0290] Simon C.B., Bishop M.D., Wallace M.R., Staud R., DelRocco N., Wu S.S., Dai Y., Borsa P.A., Greenfield W.H., Fillingim R.B., George S.Z. (2023). Circulating inflammatory biomarkers predict pain change following exercise-induced shoulder injury: findings from the biopsychosocial influence on shoulder pain preclinical trial. J. Pain.

[bb0295] Song L., Makowski G.S. (2017). Advances in Clinical Chemistry.

[bb0300] Tanaka T., Narazaki M., Kishimoto T. (2014). IL-6 in inflammation, immunity and disease. Cold Spring Harb. Perspect. Biol..

[bb0305] Testa B., Reid J., Scott M.E., Murison P.J., Bell A.M. (2021). The short form of the glasgow composite measure pain scale in post-operative analgesia studies in dogs: a scoping review. Front. Vet. Sci..

[bb0310] Tylicka M., Guszczyn T., Maksimowicz M., Kamińska J., Matuszczak E., Karpińska M., Koper-Lenkiewicz O.M. (2021). The concentration of selected inflammatory cytokines (IL-6, IL-8, CXCL5, IL-33) and damage-associated molecular patterns (HMGB-1, HSP-70) released in an early response to distal forearm fracture and the performed closed reduction with kirschner wire fixation in children. Front. Endocrinol..

[bb0315] Vali A.M., Toliyat T., Shafaghi B., Dadashzadeh S. (2008). Preparation, optimization, and characterization of topotecan loaded PEGylated liposomes using factorial design. Drug Dev. Ind. Pharm..

[bb0320] Vu M.T., Le N.T.T., Pham T.L.-B., Nguyen N.H., Nguyen D.H. (2020). Development and characterization of soy lecithin liposome as potential drug carrier systems for codelivery of letrozole and paclitaxel. J. Nanomater..

[bb0325] Wang W.X., Feng S.S., Zheng C.H. (2016). A comparison between conventional liposome and drug-cyclodextrin complex in liposome system. Int. J. Pharm..

[bb0330] Wani A.R., Roy R.K., Ashraf A., Roy D.C.J.V.W. (2013). Pharmacokinetic studies of meloxicam after its intravenous administration in local goat (Capra hircus) of Assam. Vet. World.

[bb0335] Wu I.Y., Bala S., Škalko-Basnet N., di Cagno M.P. (2019). Interpreting non-linear drug diffusion data: utilizing Korsmeyer-Peppas model to study drug release from liposomes. Eur. J. Pharm. Sci..

[bb0340] Yang X., Ricciardi B.F., Hernandez-Soria A., Shi Y., Pleshko Camacho N., Bostrom M.P. (2007). Callus mineralization and maturation are delayed during fracture healing in interleukin-6 knockout mice. Bone.

[bb0345] Yang Z., Liu L., Sheng L., Wang H., Li C., Lin X., Yang P. (2024). Design of an injectable sustained release in-situ forming depot of meloxicam for pain relief. J. Drug Deliv. Sci. Technol..

[bb0350] Yegireddy M., Nadoor P., Rao S., Hanumanthu P.B., Rajashekaraiah R., Ramachandrappa S.C., Halemani G.M., Mannem S., Prasad T., Ubaradka S. (2022). Chitosan encapsulated meloxicam nanoparticles for sustained drug delivery applications: preparation, characterization, and pharmacokinetics in wistar rats. Molecules.

[bb0355] Zeng Q., Zeng Y., Slevin M., Guo B., Shen Z., Deng B., Zhang W. (2021). C-reactive protein levels and clinical prognosis in LAA-type stroke patients: a prospective cohort study. Biomed. Res. Int..

[bb0360] Zhao L., Wang D., Yu J., Wang X., Wang T., Yu D., Elfalleh W. (2024). Complex phospholipid liposomes co-encapsulated of proanthocyanidins and α-tocopherol: Stability, antioxidant activity and in vitro digestion simulation. Food Biosci..

